# Triclabendazole Induces Pyroptosis by Activating Caspase-3 to Cleave GSDME in Breast Cancer Cells

**DOI:** 10.3389/fphar.2021.670081

**Published:** 2021-07-08

**Authors:** Liang Yan, Yi Liu, Xue-feng Ma, Dan Hou, Yu-hui Zhang, Yong Sun, Shan-shan Shi, Tim Forouzanfar, Hai-yan Lin, Jun Fan, Gang Wu

**Affiliations:** ^1^ Department of Medical Biochemistry and Molecular Biology, School of Medicine, Jinan University, Guangzhou, China; ^2^ Guangzhou Key Laboratory of Basic and Applied Research of Oral Regenerative Medicine, Affiliated Stomatology Hospital of Guangzhou Medical University, Guangzhou, China; ^3^ Department of Obstetrics and Gynecology, The First Affiliated Hospital of Jinan University, Guangzhou, China; ^4^ Department of Oral and Maxillofacial Surgery/Pathology, Amsterdam UMC/VUmc Location and Academic Centre for Dentistry Amsterdam (ACTA), Vrije Universitetit Amsterdam, Amsterdam Movement Science, Amsterdam, Netherlands; ^5^ Savaid Stomatology School, Hangzhou Medical College, Hangzhou, China; ^6^ Department of Oral Implantology and Prosthetic Dentistry, Academic Centre of Dentistry Amsterdam (ACTA), Universiteit van Amsterdam and Vrije Universiteit Amsterdam, Amsterdam, Netherlands

**Keywords:** triclabendazole, pyroptosis, gasdermin E, caspase-3, breast cancer

## Abstract

Pyroptosis is a form of programmed cell death, in which gasdermin E (GSDME) plays an important role in cancer cells, which can be induced by activated caspase-3 on apoptotic stimulation. Triclabendazole is a new type of imidazole in fluke resistance and has been approved by the FDA for the treatment of fascioliasis and its functions partially acting through apoptosis-related mechanisms. However, it remains unclear whether triclabendazole has obvious anti-cancer effects on breast cancer cells. In this study, to test the function of triclabendazole on breast cancer, we treated breast cancer cells with triclabendazole and found that triclabendazole induced lytic cell death in MCF-7 and MDA-MB-231, and the dying cells became swollen with evident large bubbles, a typical sign of pyroptosis. Triclabendazole activates apoptosis by regulating the apoptoic protein levels including Bax, Bcl-2, and enhanced cleavage of caspase-8/9/3/7 and PARP. In addition, enhanced cleavage of GSDME was also observed, which indicates the secondary necrosis/pyroptosis is further induced by active caspase-3. Consistent with this, triclabendazole-induced GSDME–N-terminal fragment cleavage and pyroptosis were reduced by caspase-3–specific inhibitor (Ac-DEVD-CHO) treatment. Moreover, triclabendazole induced reactive oxygen species (ROS) elevation and increased JNK phosphorylation and lytic cell death, which could be rescued by the ROS scavenger (NAC), suggesting that triclabendazole-induced GSDME-dependent pyroptosis is related to the ROS/JNK/Bax-mitochondrial apoptotic pathway. Besides, we showed that triclabendazole significantly reduced the tumor volume by promoting the cleavage of caspase-3, PARP, and GSDME in the xenograft model. Altogether, our results revealed that triclabendazole induces GSDME-dependent pyroptosis by caspase-3 activation at least partly through augmenting the ROS/JNK/Bax-mitochondrial apoptotic pathway, providing insights into this on-the-market drug in its potential new application in cancer treatment.

## Introduction

Breast cancer is the most frequent cancer among women (Anastasiadi et al., 2017; Tong et al., 2018; Saneei Totmaj et al., 2019), affecting 2.1 million women each year. Breast cancer causes the greatest number (approximately 15%) of cancer-related deaths among women (Bray et al., 2018). In addition to surgery, chemotherapy, which targets and destroys breast cancer cells, is often used to control/shrink larger tumors and prevent reoccurrence. Most of the anti-cancer therapies trigger apoptosis—a type of programmed cell death (PCD) (Ouyang et al., 2012)—and related cell death networks to eliminate malignant cells. Although chemotherapy is the main strategy for a variety of cancers, the overall response rate of chemotherapy in breast cancer patients remains unsatisfactory. One major mechanism accounting for such a drug resistance is the development and activation of anti-apoptotic systems, allowing cancer cells to escape drug-induced apoptosis (Takagi et al., 2015). One potential approach is to introduce novel drugs that can trigger non-apoptotic PCD.

Pyroptosis, one form of non-apoptotic PCD (also named programmed necrosis) (Robinson et al., 2019), has been recently indicated to play an important role in chemotherapy for cancers. Pyroptosis was initially regarded as a general inflammation response for innate immunity in vertebrates (Shi et al., 2017; Robinson et al., 2019). Recent studies indicated that chemotherapy drug–activated caspase-3 can also induce secondary necrosis/pyroptosis in both cancer and normal cells with expression of gasdermin E (GSDME), a member of the gasdermin family (Rogers et al., 2017; Wang et al., 2017). Pyroptosis is featured by pore formation on the plasma membrane, thereby causing disruption of cell osmotic barrier and subsequent cell swelling (Robinson et al., 2019). When apoptosis initiates, the active caspase-3 cleaves GSDME to generate the N-terminal fragment of GSDME (GSDME-NT). GSDME-NT will translocate to and perforate the plasma membrane, leading to pyroptosis (Coleman et al., 2001; Rogers et al., 2017; Wang et al., 2017). Consequently, drugs that cause pyroptosis of cancer cells can be a good supplement to conventional apoptotic PCD-based anti-cancer drugs.

Triclabendazole, a type of imidazole, has been the drug for treating liver fluke infections in livestock for over 20 years and used successfully to treat human cases of fascioliasis (Fairweather, 2005; Fairweather, 2009). Triclabendazole takes effect possibly by making tubulin polymerization and mitotic activity interfere (Robinson et al., 2002); it also inhibits adenylate cyclase activity or the association of GTP-Ras with adenylate cyclase (Lee et al., 2013) and induces DNA strand breaking and apoptosis in the reproductive organs of flukes (Hanna et al., 2013). Furthermore, triclabendazole and its metabolites can inhibit ABCG2/BCRP, an ATP-binding cassette transporter that extrudes compounds from cells in the intestine, liver, kidney, and gland, affecting pharmacokinetics of anti-cancer drugs (Barrera et al., 2012). In addition, triclabendazole possesses similar chemical structures to chemotherapy drugs, including BAY-1816032, fenbendazole, albendazole, and mebendazole (Zhou et al., 2017; Dogra et al., 2018; Siemeister et al., 2019; Williamson et al., 2020). These properties confer triclabendazole as a great potential to treat cancers. However, no study on its anti-cancer effect and potential mechanisms has been reported yet.

In this study, we used breast cancer MCF-7 and MDA-MB-231 cell lines as a cellular model, which have been shown to express GSDME protein (Wang et al., 2017), to explore the effects of triclabendazole on cancer by inducing GSDME-dependent pyroptosis via activating caspase-3. Our data showed that triclabendazole could induce apoptosis and secondary necrosis/pyroptosis by caspase-3 activation at least partly through augmenting the ROS/JNK/Bax-mitochondrial apoptotic pathway, and it also reduced the tumor volume in breast cancer cells by inducing apoptosis-to-pyroptosis. The finding suggested that triclabendazole had a therapeutic potential to treat cancer by inducing apoptosis-to-pyroptosis.

## Materials and Methods

### Reagents

Triclabendazole, Hoechst 33342, propidium iodide (PI), and dimethyl sulfoxide (DMSO) were purchased from Sigma-Aldrich (St. Louis, MO, United States). Triclabendazole was dissolved in DMSO at 100 mM and stored at -20°C. Dulbecco’s modified Eagle’s medium (DMEM), fetal bovine serum (FBS), streptomycin, and penicillin were obtained from Thermo/Fisher/Invitrogen (Carlsbad, CA, United States). Ac-DEVD-CHO (#HY-P1001) was purchased from MedChemExpress (Princeton, NJ, United States). The antibody against DFNA5/GSDME (#ab215191) was the product of Abcam (Cambridge, United Kingdom). The antibodies against Bcl-2 (#4223), Bax (#2772), cleaved caspase-3 (#9661), cleaved caspase-7 (#12827), cleaved caspase-8 (#9496), caspase-9 (#9508), PARP (#9542), β-actin (#8457), and horseradish peroxidase (HRP)-conjugated goat anti-rabbit IgG (#7074) were obtained from Cell Signaling Technology (Danvers, MA, United States). The annexin V-FITC/PI apoptosis assay kit (#BB4101) and mitochondrial membrane potential assay kit with JC-1 (#BB-4105-2) were from BestBio (Shanghai, China).

### Cell Culture

The human breast cancer cell lines (MCF-7 and MDA-MB-231) were obtained from the Shanghai Cell Bank of Type Culture Collection of Chinese Academy of Sciences (Shanghai, China). The cells were cultured in complete DMEM (containing 10% FBS, 100 IU/ml penicillin, 100 μg/ml streptomycin, and 2 mM L-glutamine) at 37°C in a humidified incubator with 5% CO_2_ and sub-cultured every 2–3 days.

### Cell Viability Assay

Cell viability was detected by the CCK-8 assay. The cells were seeded in 96-well plates and then treated with indicated concentrations of triclabendazole for 24 h or 48 h. The cells were co-incubated with CCK-8 solution for 1 h at 37°C. The optical density (OD) values were measured at 450 nm by using a Varioskan Flash microplate reader (Thermo Fisher Scientific, United States).

### Flow Cytometry

For annexin V-FITC/PI double staining, the cells were harvested and washed twice with cold PBS, and then the cells were stained with annexin V in binding buffer for 15 min at room temperature, followed by staining with PI for 5 min without annexin V. The cells were then analyzed by flow cytometry using the BD FACSCanto II Flow Cytometer (FACSCanto; Becton Dickinson). Data were acquired and analyzed by using the CELLQuest software (Becton Dickinson).

### Cell Death Assay

Lytic cell death was measured by PI incorporation as described previously (Py et al., 2014; Li et al., 2017). The cells were seeded in glass-bottomed dishes and then treated with indicated concentrations of triclabendazole for 24 h. The cells were stained with PI solution (2 μg/ml PI plus 5 μg/ml Hoechst 33342) for 10 min at room temperature and were observed immediately by live imaging using the Leica ATCSSP8 Confocal Microscope (Leica, Germany).

### Mitochondrial Membrane Potential Measurement

The mitochondrial membrane potential assay kit with JC-1 was used to measure the mitochondrial membrane potential, according to the manufacturer’s instructions. The cells were stained with JC-1 working solution for 15 min at 37 °C, washed twice with JC-1 staining buffer, and observed under the Leica ATCSSP8 Confocal Microscope. The ratio of the JC-1 aggregate to monomer was analyzed by the Leica software.

### Measurement of ROS

The ROS levels were measured by an ROS Assay Kit with DCFH-DA (BestBio, Shanghai, China) according to the manufacturer's instructions. The cells were stained with DCFH-DA working solution for 20 min at 37 °C and then washed three times with PBS. Fluorescence images were captured by a Leica ATCSSP8 Confocal Microscope, and the relative fluorescence unit was analyzed by the Leica software.

### Western Blot Analysis

Western blotting was performed essentially as previously described (Li et al., 2017). The proteins were dissolved with 1× loading buffer and separated by SDS-PAGE and then electro-transferred to PVDF membranes (Hybond-P; GE Healthcare Life Sciences, Piscataway, NJ, United States). The membranes were blocked in blocking buffer and then incubated with the primary antibody overnight at 4°C, followed by incubation with the secondary antibody for 1 h at room temperature. The blot images were captured by the Molecular Imager^®^ Gel Doc™ XR^+^ imaging system (Bio-Rad, United States).

### Nude Mouse Xenograft Assay

Female nude mice (BALB/c, 4 weeks old) were bought from the Experimental Animal Center of Southern Medical University (Guangzhou, China), and they were acclimatized under 12 h dark/12 h light cycles for 1 week before experiments. All animal experiments were approved by the Animal Ethics Committee of Southern Medical University (Resolution No. L2018153).

The mouse xenograft model was established as previously described (Aka et al., 2009). A total of 1 × 10^6^ breast cancer MDA-MB-231 cells were injected subcutaneously into the right flanks of mice. The mice were maintained and housed under specific pathogen–free conditions. When the tumor volumes reached approximately 100 mm^3^, the mice were divided randomly into three treatment groups (*n* = 6) and intraperitoneally injected twice a week with triclabendazole solution (20 or 100 mg/ kg body weight) or vehicle (5% ethanol in PBS). The length and width of the tumor were monitored twice a week using calipers. The tumor volume (V) was calculated as follows: V = [(length) × (width) × (width)]/2. At the end of the experiment, the mice were sacrificed, and the xenograft tumors were measured. The markers of apoptosis and pyroptosis in the xenograft tumors were detected by western blot.

### Immunohistochemical Analysis

After routine deparaffinization and hydration, the tissue sections from mouse tumor tissue specimens of each group were boiled in a microwave oven for 10 min for antigen retrieval followed by washing three times with PBS (pH 7.4). Then, they were treated with 3% hydrogen peroxide for 25 min and washed three times with PBS (pH 7.4). Subsequently, the tissue sections were blocked in blocking buffer containing 3% BSA and incubated with anti-cleaved caspase-3 antibodies overnight at 4 °C followed by incubating with the secondary antibody and DAB. The sections were counterstained using hematoxylin for 3 min and washed under tap water. The images were captured by the Leica microscope.

### Statistical Analysis

Each experiment was performed three times independently. Data were presented as mean ± SD. Statistical analysis was performed using GraphPad Prism 5.0. One-way analysis of variance (ANOVA) followed by Tukey’s post hoc test and unpaired Student’s *t*-test was used to analyze the statistical significance among the groups. *p <* 0.05 was considered statistically significant (ns, *p >* 0.05; *p < 0.05; **p < 0.01; ***p < 0.001).

## Results

### Triclabendazole Induced Apoptosis and Lytic Cell Death in Breast Cancer Cells by Reducing the Mitochondrial Membrane Potential

**FIGURE 1:**
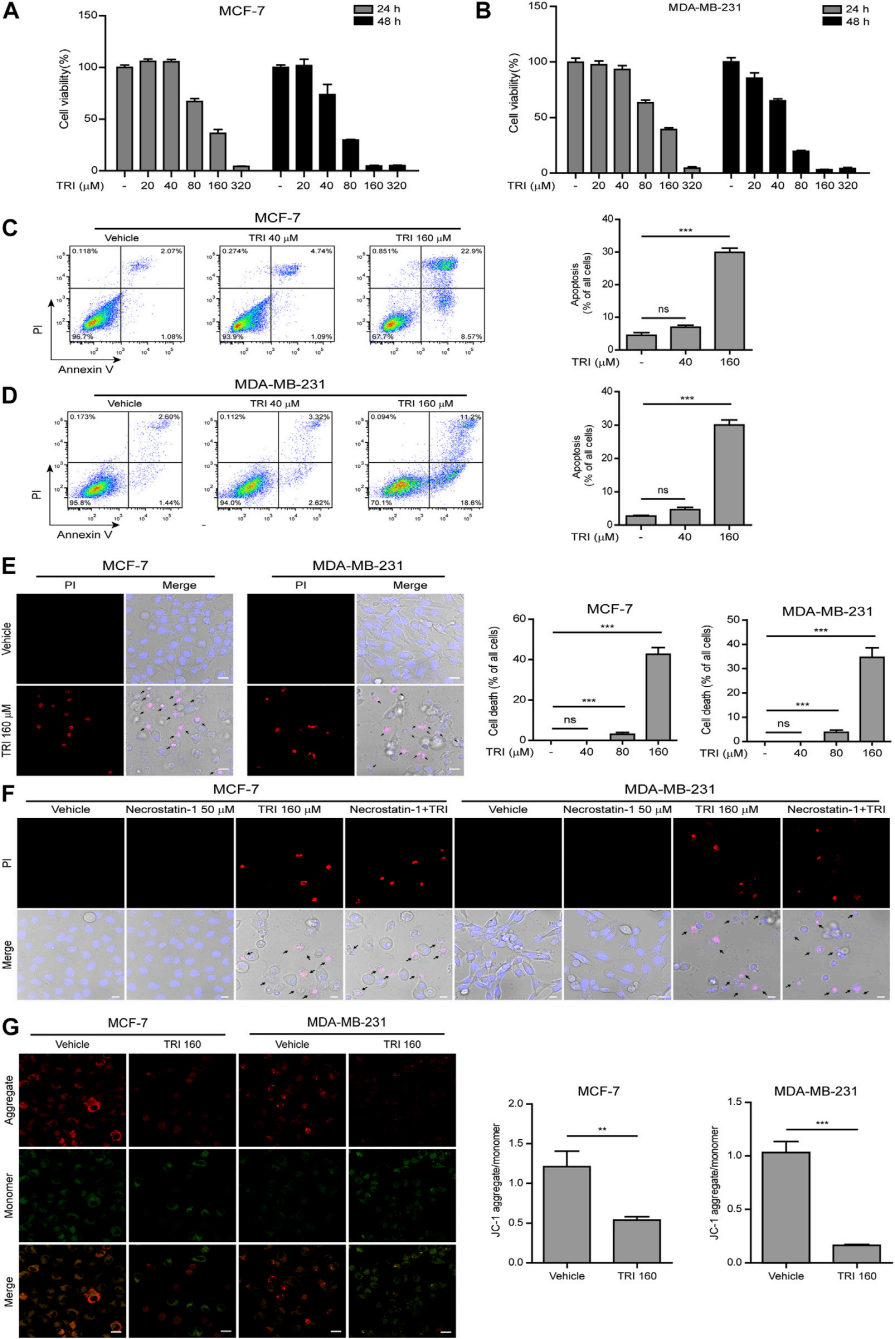
Triclabendazole induces apoptosis and lytic death in breast cancer cells. **(A, B)** The cell viability is measured by the CCK-8 assay. The MCF-7 **(C)** or MDA-MB-231 **(D)** cells are treated with indicated concentrations of triclabendazole (TRI) for 24 h. The cells are stained with annexin V-FITC and PI and then analyzed by flow cytometry. The ratios of cells are shown in each quadrant of representative dot plots of flow cytometry (on the left), and quantitative analysis of the ratios of apoptotic cells and necrotic/pyroptotic cells is shown on the right. Data are shown as mean ± SD (*n* = 3). **(E, F)** The cells are treated as in **(C, D)**, respectively, stained with PI solution containing 2 μg/ ml propidium iodide (PI; red, staining dying cells) plus 5 μg/ ml Hoechst 33342 (blue, staining all cells) for 10 min, and then observed by the Leica confocal microscope; the black arrow indicates lytic cells (necrotic or pyroptotic) with large bubbles blowing from the cellular membrane. Scale bars: 10 µm. **(G)** The mitochondrial membrane potential is determined by the JC-1 assay. The cells are observed by the Leica confocal microscope, and representative images are shown. Scale bars: 10 µm. Statistical analysis is performed by one-way ANOVA with Tukey’s multiple comparisons test. **p < 0.01; ***p < 0.001; ns, not significant; TRI, triclabendazole.

To determine effects of triclabendazole on breast cancer, firstly, we applied the CCK-8 assay to measure the cytotoxicity on MCF-7 and MDA-MB-231 cells. Our data showed that triclabendazole (160 µM) could significantly decrease the metabolic activity of breast cancer cells (Figures 1A,B). We further adopted the annexin V-FITC/PI assay to analyze whether triclabendazole induced apoptosis of breast cancer cells. Triclabendazole at 160 µM induced significant apoptosis (annexin V^+^/PI^−^) and lytic death (annexin V^+^/PI^+^) in both cells (Figures 1C,D). Furthermore, the result of PI solution staining showed that triclabendazole could induce significant lytic cell death of about 40% in MCF-7 cells and 35% in MDA-MB-231 cells (Figure 1E). Meanwhile, the cells treated with triclabendazole became round and swollen with evident large bubbles extending from the plasma membrane, which is regarded as a typical characteristic of secondary necrosis/pyroptosis different from apoptosis (Figure 1E). We further showed the triclabendazole-induced lytic cell death could not be inhibited by the pre-treatment of necrostatin-1, a necrosis inhibitor (Figure 1F), which suggested that triclabendazole induced pyroptosis but not necrosis in breast cancer cells.

Disrupting mitochondrial function is closely related to apoptosis, and triclabendazole is a potent apoptotic anti-fluke drug (Hanna et al., 2013). We sought to further explore whether triclabendazole induced apoptosis in breast cancer cells through disrupting mitochondrial function. The change of mitochondrial membrane potential in MCF-7 and MB-MDA-231 cells after triclabendazole treatment was examined using JC-1 staining, and we observed that triclabendazole significantly decreased the ratio of JC-1 aggregates (red) to monomers (green), indicating a reduction of membrane potential in both cells after triclabendazole treatment (Figure 1G).

Together, these results indicated that the reduction of the mitochondrial membrane potential was involved in triclabendazole-induced apoptosis and lytic cell death/pyroptosis in breast cancer cells.

### Apoptotic Pathways Mediate Triclabendazole-Induced Pyroptosis in Breast Cancer Cells

**FIGURE 2:**
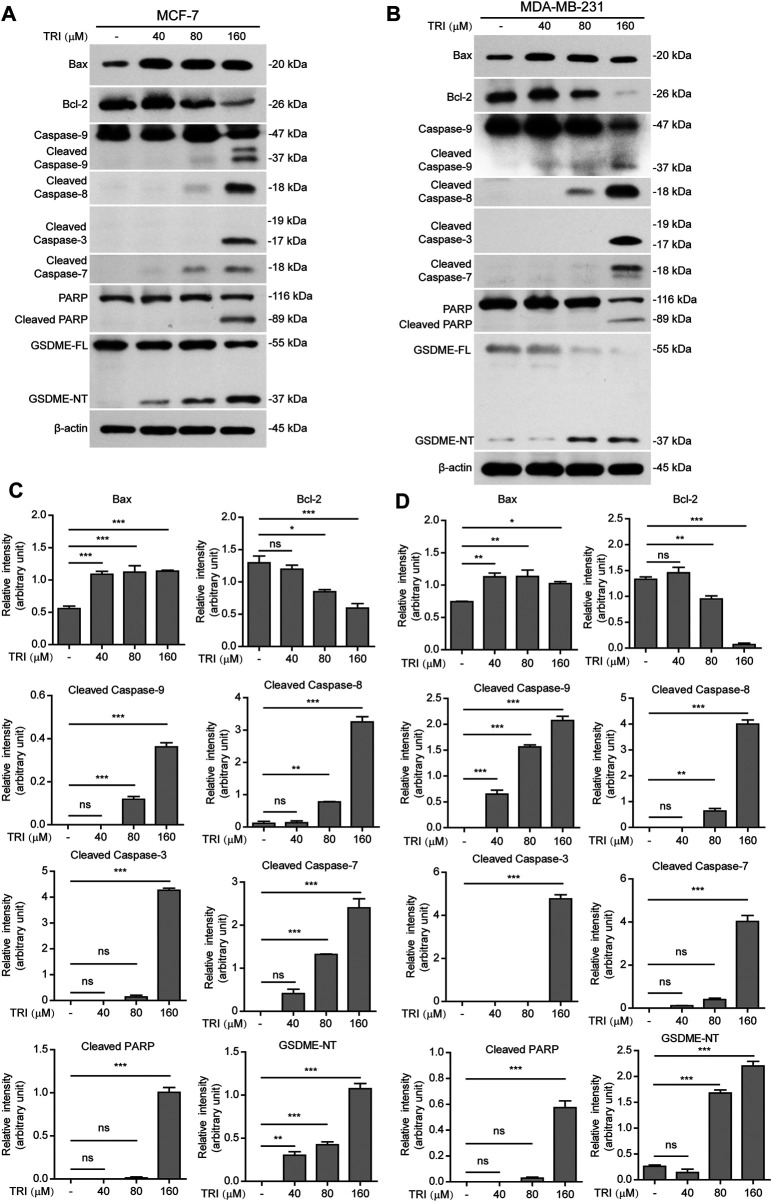
Western blot analysis of apoptotic and lytic cell death markers in breast cancer cells treated with triclabendazole. MCF-7 and MDA-MB-231 cells are treated with indicated concentrations of triclabendazole for 24 h. **(A, B)** Markers of apoptosis and lytic cell death are detected by western blotting. The β-actin is recruited as a loading control. **(C, D)** Relative gray values of Bax, Bcl-2, cleaved caspase-9, cleaved caspase-8, cleaved caspase-3, cleaved caspase-7, cleaved PARP, and GSDME-NT blots are quantified in **(A, B),** respectively. Data are shown as mean ± SD (*n* = 3). Statistical analysis is performed by one-way ANOVA with Tukey’s multiple comparisons test. *p < 0.05; **p < 0.01; ***p < 0.001; ns, not significant; TRI, triclabendazole.

To further investigate the underlying mechanisms of triclabendazole-induced apoptosis and lytic cell death in breast cancer cells, we next analyzed the protein markers of apoptosis by immunoblotting, including Bax, Bcl-2, cleaved caspase-9, cleaved caspase-8, cleaved caspase-3, cleaved caspase-7, and cleaved PARP. As expected, our results showed that triclabendazole treatment up-regulated the expression of Bax and down-regulated the expression of Bcl-2 in the cell lysates (Figure 2). Moreover, the apoptotic initiator caspase-8 and caspase-9 were activated and cleaved in triclabendazole-treated cells in a dose-dependent manner. Consistent with this, the downstream executioner caspase-3 and caspase-7 were correspondingly activated and processed to their cleaved forms. Consequently, poly (ADP-ribose) polymerase (PARP) was cleaved to produce the carboxy-terminal catalytic domain (89 kDa) (Figure 2). The above results indicated both intrinsic and extrinsic apoptotic pathways were initiated.

Recently, gasdermin E (GSDME) has been shown to execute pyroptosis by forming pores in the cell membranes through the GSDME-N domains (37 kDa), which are cleaved from GSDME by cleaved caspase-3, assembling into polymers to bind to cell membranes under intrinsic and extrinsic apoptotic pathways (Rogers et al., 2017; Wang et al., 2017). Therefore, we next explored whether GSDME was expressed and cleaved to form GSDME-N domains in the triclabendazole-treated cells. Upon triclabendazole treatment, GSDME was found to be cleaved in both MCF-7 and MDA-MB-231 cells (Figure 2), which was correlated with the activation levels of caspase-3 and the levels of lytic cell death. These results suggested that triclabendazole induced lytic cell death through GSDME activation.

### Cleaved Caspase-3 Is the Key Switch for GSDME-Mediated Cell Death in Breast Cancer Cells Treated With Triclabendazole

**FIGURE 3:**
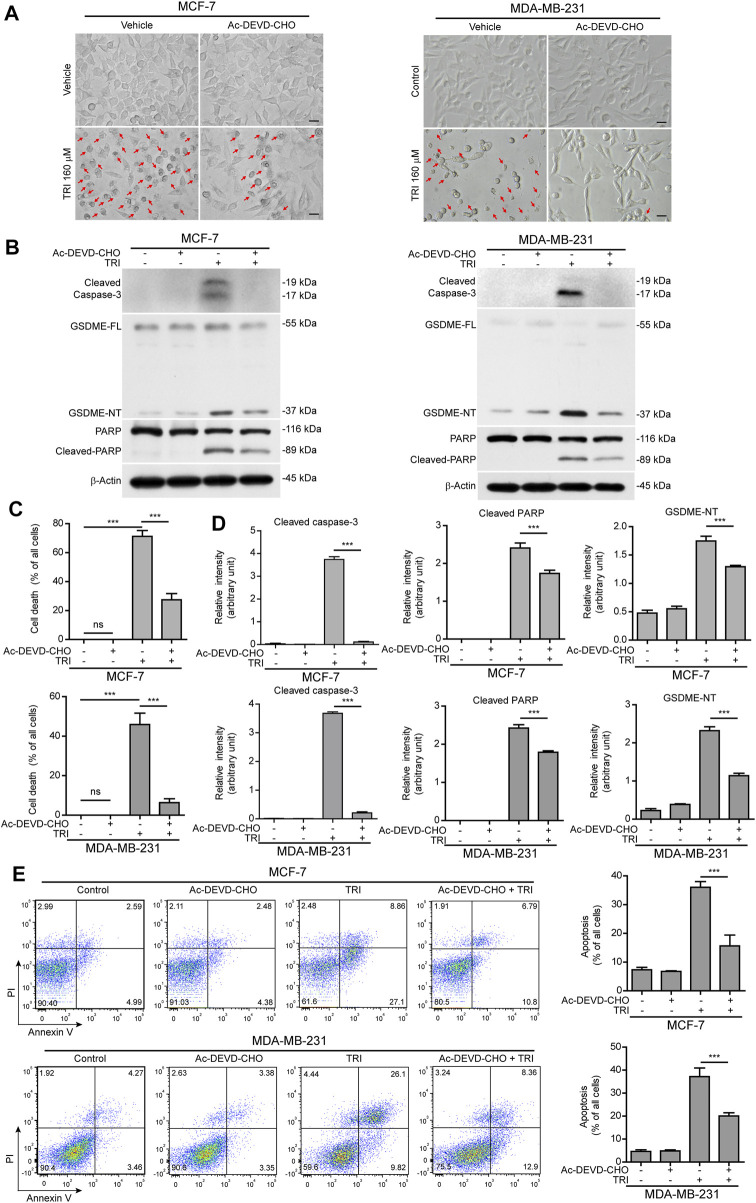
Blockade of caspase-3 activity suppresses triclabendazole-induced lytic cell death in breast cancer cells. MCF-7 and MDA-MB-231 cells are pre-treated with the caspase-3 inhibitor Ac-DEVD-CHO (DEVD) for 1 h and then treated with triclabendazole for 24 h. **(A)** The cells are observed immediately by live imaging using fluorescence microscopy, and representative bright-field images are shown. The red arrow indicates lytic cells (necrotic or pyroptotic) with large bubbles blowing from the cellular membrane. Scale bars: 20 µm. **(B)** Representative western blots for markers of apoptosis and lytic cell death including cleaved caspase-3, cleaved PARP, and GSDME-NT. The β-actin is recruited as a loading control. **(C)** The ratio of lytic cell death (necrosis or pyroptosis) in five randomly chosen fields with each containing ∼50 cells is quantified to **(A)**. Data are shown as mean ± SD (*n* = 5). **(D)** Histograms showing the relative gray values of cleaved PARP and GSDME-NT in **(B)**. **(E)** Cells are stained with annexin V-FITC and PI and then analyzed by flow cytometry. Quantitative analysis of the ratios of apoptotic cells and necrotic/pyroptotic cells is shown on the right. Statistical analysis is performed by one-way ANOVA with Tukey’s multiple comparisons test. ***p < 0.001; ns, not significant; TRI, triclabendazole.

Since cleaved caspase-3 is the key enzyme to process GSDME into the GSDME-NT (37 kDa) fragment (Rogers et al., 2017; Wang et al., 2017), we next explored whether blocking caspase-3 activation would attenuate triclabendazole-induced cell death. MCF-7 and MDA-MB-231 cells were treated with triclabendazole in the presence or absence of the caspase-3–specific inhibitor Ac-DEVD-CHO (DEVD). As shown in Figure 3, DEVD pre-treatment could attenuate triclabendazole-induced cell death. Consistent with this, DEVD pre-treatment significantly suppressed the levels of cleaved caspase-3 (17/19 kDa), cleaved GSDME (37 kDa), and cleaved PARP (89 kDa) formation. In addition, DEVD pre-treatment also inhibited triclabendazole-induced apoptosis by flow cytometry detection and reduced lytic cell death (Figure 3), even though inhibition of caspase-3 did not reduce the levels of ROS and recover the mitochondrial membrane potential (data not shown). Together, these results indicated that caspase-3–mediated cleavage of GSDME contributed to triclabendazole-induced lytic cell death and that the levels of GSDME-NT were correlated with the lytic cell death.

### Triclabendazole-Induced GSDME-Dependent Pyroptosis Is Downstream of the ROS/JNK/Bax-Mitochondrial Apoptotic Pathway

**FIGURE 4:**
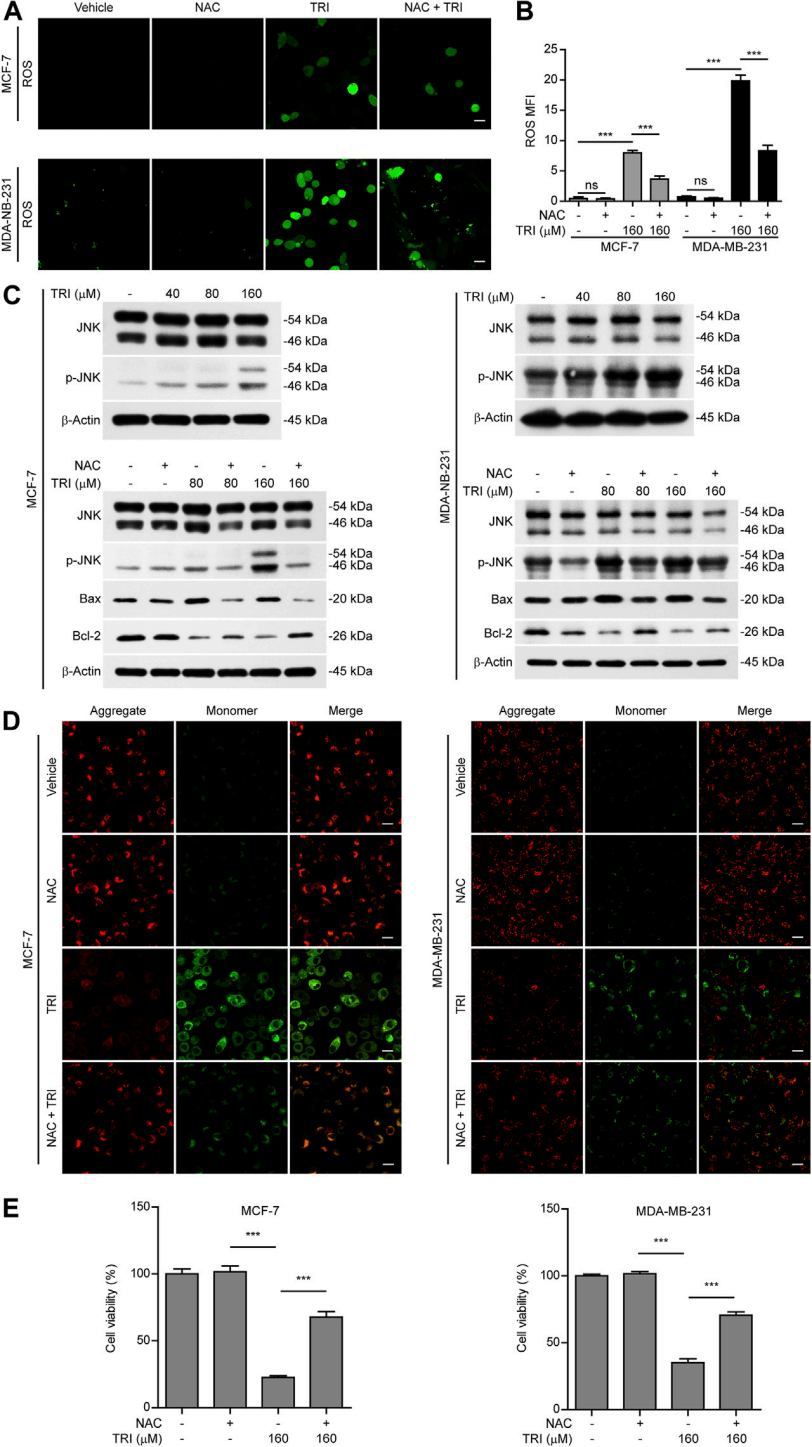
Triclabendazole enhances the ROS/JNK/Bax-mitochondrial pathway. The breast cancer cells (MCF-7 or MDA-MB-231) are pre-treated with or without 5 mM NAC (an antioxidant) followed by co-incubation with triclabendazole for 24 h. **(A)** The ROS levels in MCF-7 and MDA-MB-231 are measured by a DCFH-DA probe. The cells are observed, and representative images are shown. Scale bars: 10 μm, *n* = 3. **(B)** Relative ROS levels analyzed by the Leica software in ten randomly chosen fields with each containing ∼40 cells in **(A)**; data are shown as mean ± SD (*n* = 10). **(C)** Representative western blots for proteins levels and protein phosphorylation levels including Bax, Bcl-2, JNK, and *p*-JNK. **(D)** The mitochondrial membrane potential is determined by the JC-1 assay. The cells are observed by the Leica confocal microscope, and representative images are shown. Scale bars: 10 µm. **(E)** The cell viability is measured by the CCK-8 assay in MCF-7 and MDA-MB-231 cells with/without NAC treatment, respectively. Statistical analysis is performed by one-way ANOVA with Tukey's multiple comparisons test. ***p < 0.001; ns, not significant; TRI, triclabendazole.

In light of previous findings that ROS/JNK signaling was important for the mitochondrial apoptotic pathway and the above-mentioned results showing that triclabendazole up-regulated Bax expression and initiated the intrinsic apoptotic pathway, we next explored whether triclabendazole induced pyroptosis through the ROS/JNK/Bax-mitochondrial apoptotic pathway. We determined ROS levels in breast cancer cells treated with triclabendazole and found that the levels of ROS in MCF-7 and MDA-MB-231 increased after triclabendazole treatment (Figures 4A,B). Meanwhile, we observed that the phosphorylation of JNK increased significantly by triclabendazole treatment but did not change the constitutive expression of JNK (Figure 4C).

**FIGURE 5:**
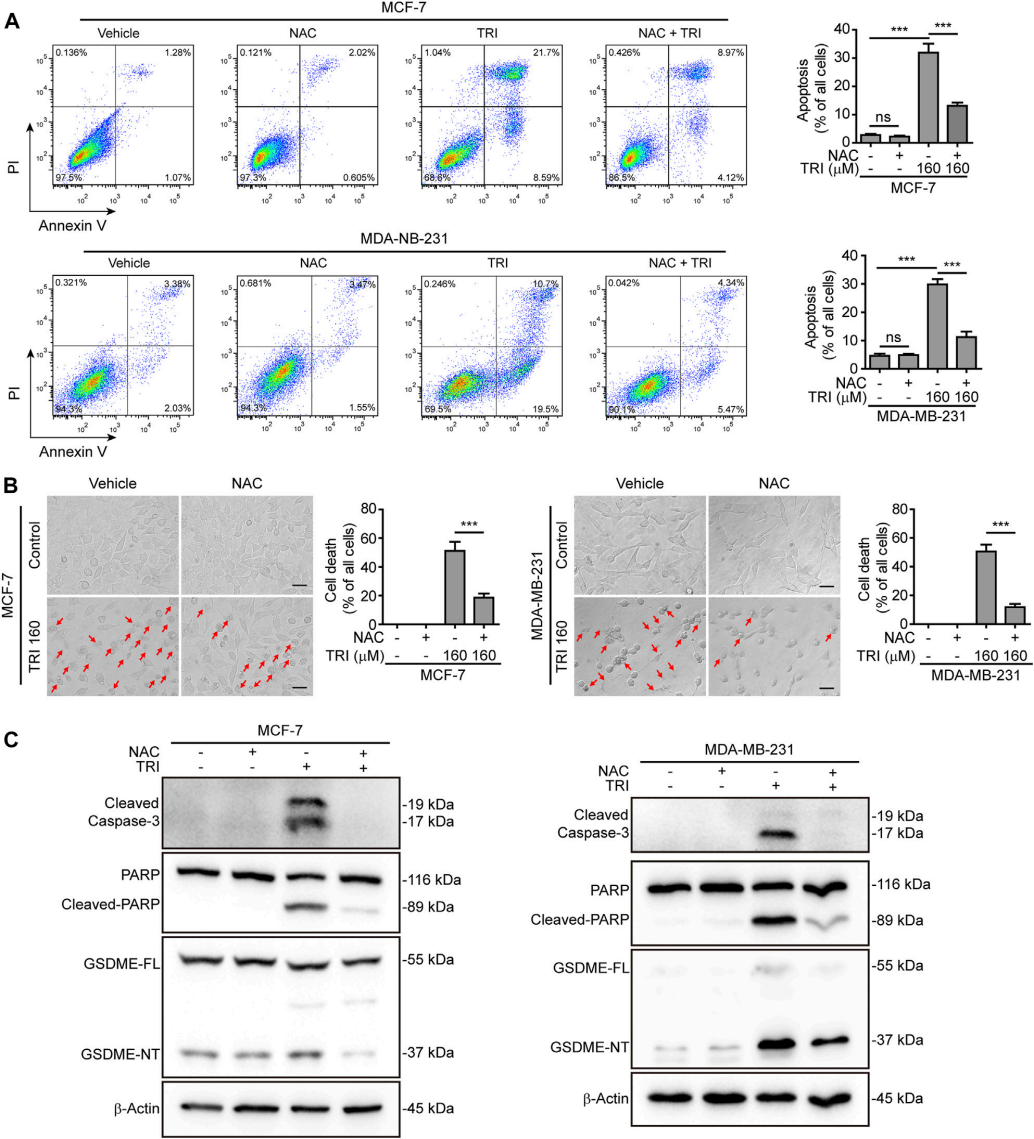
Triclabendazole-induced apoptosis and lytic cell death are reversed by NAC treatment. **(A)** The cells are analyzed by flow cytometry, and numbers represent the ratios of cells in each quadrant. **(B)** The cells are observed immediately by live imaging using fluorescence microscopy, and representative bright-field images are shown (*n* = 5). Scale bars: 20 µm. **(C)** Western blots are used to detect the protein levels of cleaved caspase-3, cleaved PARP, and GSDME-NT, respectively. The β-actin is recruited as a loading control. Statistical analysis is performed by one-way ANOVA with Tukey’s multiple comparisons test. ***p < 0.001; ns, not significant; TRI, triclabendazole.

To further explore the role of ROS and JNK signaling in pyroptosis upon triclabendazole treatment, we used NAC, a reactive oxygen scavenger, to pre-treat breast cancer cells and then co-incubated with triclabendazole. The results showed that NAC markedly inhibited the ROS elevation induced by triclabendazole (Figures 4A,B). We also found that the effects of triclabendazole on the expression of Bax and Bcl-2 and the phosphorylation of JNK were partly antagonized by NAC pre-treatment (Figure 4C). Consistent with these results, we observed that NAC could significantly reverse the decrease of triclabendazole-induced ratio of JC-1 aggregates (red) to monomers (green), and it also could improve the cell viability (Figures 4D,E). In addition, flow cytometry and live imaging assay analysis showed that triclabendazole-induced lytic cell death was also significantly reduced by NAC treatment as compared with control, in which the number of cells with large bubbles blowing from the plasma membrane and apoptotic morphology reduced (Figures 5A,B). Similarly, NAC pre-treatment significantly suppressed the levels of cleaved caspase-3 (17/19 kDa), cleaved GSDME (37 kDa), and cleaved PARP (89 kDa) formation (Figure 5C). These results revealed that triclabendazole induced pyroptosis at least partly through augmenting the ROS/JNK/Bax-mitochondrial apoptotic pathway.

### Triclabendazole Induces Apoptosis-to-Pyroptosis in a Xenograft Model

**FIGURE 6:**
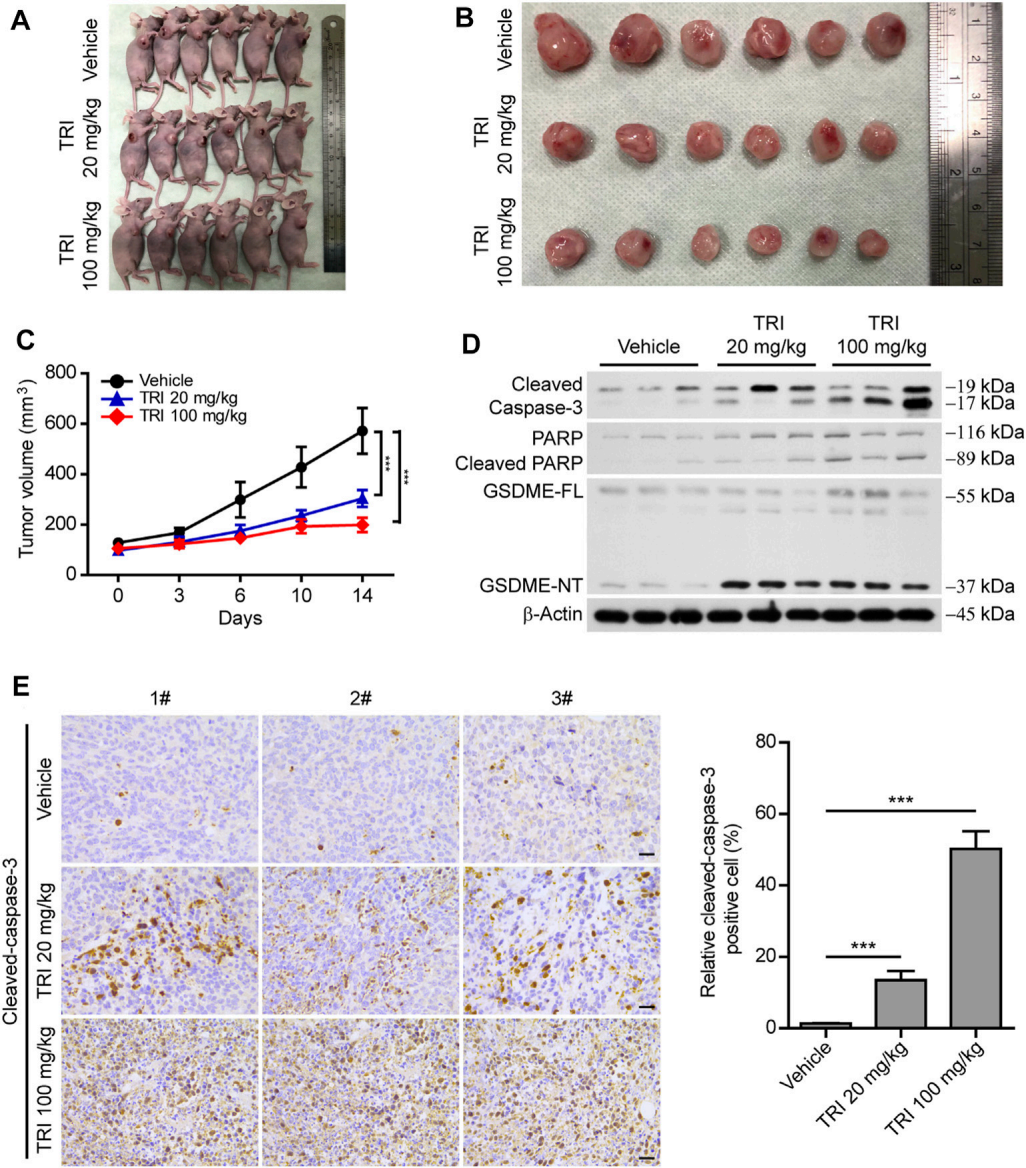
Anti-tumor effect of triclabendazole in the xenograft model. BALB/C nude mice are injected subcutaneously with 1 × 10^6^ MDA-MB-231 breast cancer cells into their right flanks and then injected intraperitoneally with triclabendazole (20 mg/kg or 100 mg/kg) or vehicle. **(A)** Tumor in BALB/C nude mice. **(B)** Image of MDA-MB-231–derived xenograft tumors at the end of the study. The nude mice are sacrificed after 2 weeks of treatment with triclabendazole, and then the tumors are carefully dissected and photographed (*n* = 6). **(C)** Curves of tumor size. Data are shown as mean ± SD (*n* = 6); statistical analysis is performed by two-way ANOVA. **(D)** Western blot analyzing protein expression of cleaved caspase-3, cleaved PARP, and GSDME in tumors derived from treatment with triclabendazole or vehicle. **(E)** Immunohistochemical analysis of protein expression of cleaved caspase-3 in tumor sections (*n* = 3); images are captured by the Leica microscope, and representative images are shown. Scale bars: 20 μm. ***p < 0.001; TRI, triclabendazole.

As the above-mentioned results showed that triclabendazole induced pyroptosis by cleaved caspase-3 *in vitro*, we further explored whether triclabendazole reduces the tumor volume *in vivo*; MDA-MB-231 breast cancer cells were implanted subcutaneously into the right flanks of BALB/C nude mice. When the mouse xenograft tumors grew to ∼100 mm^3^, nude mice were treated with triclabendazole solution (20 or 100 mg/ kg body weight) or vehicle twice a week by intraperitoneal injection. The results showed that the volume of the xenograft tumors treated with triclabendazole was significantly smaller than that treated with vehicle (Figures 6A–C), but the body weight of mice showed no difference between triclabendazole solution treatment and vehicle (data not shown). Moreover, triclabendazole administration significantly promoted formation of cleaved caspase-3, cleaved PARP, and GSDME-NT (Figure 6D), indicating that triclabendazole induced apoptosis-to-pyroptosis, which was consistent with the *in vitro* studies showing the effects of triclabendazole on breast cancer cells. In addition, the stronger staining of apoptotic protein cleaved caspase-3 was also observed in the triclabendazole treatment group by immunohistochemical analysis (Figure 6E). Therefore, these results highlight that triclabendazole treatment could reduce the tumor volume by inducing apoptosis-to-pyroptosis in the mouse xenograft model.

## Discussion

Chemotherapy is the major strategy for cancer treatment. Currently, multiple chemotherapy drugs were used in anti-cancer activities, including platinum (Decatris et al., 2004), nitrogen mustard (Lang et al., 2020), and triazole (Song et al., 2020). Recent studies reveal that, apart from executing apoptosis, chemotherapy drug–activated caspase-3 can also induce secondary necrosis/pyroptosis in cancer cells. Benzimidazole derivatives have multiple biological activities, such as anti-parasitic, anti-viral, and anti-cancer, especially on various cancer cells with high cytotoxicity (Barot et al., 2013; Błaszczak-Świątkiewicz and Mikiciuk-Olasik, 2015). Trichlorobendazole is a benzimidazole compound, but it has remained unclear whether triclabendazole has a certain pharmacological effect on cancer therapy and its underlying mechanism. Interestingly, in this study, we found that triclabendazole significantly induces apoptosis in breast cancer cells typically swollen with evident large bubbles, including positive for annexin V staining, irregulation of Bax and Bcl-2, activation of caspase-3/7/8/9, and cleavage of PARP. More worthy of attention is that many triclabendazole-treated cells became swollen with evident large bubbles extending from the plasma membrane accompanied by the generation of the GSDME-N fragment (37 kDa), suggestive of inducing pyroptosis. The effects of triclabendazole on breast cancer cells could partly reverse by a reactive oxygen scavenger NAC and caspase-3–specific inhibitor Ac-DEVD-CHO, which suggests triclabendazole induced pyroptosis through increasing the levels of ROS and activating caspase-3. In line with the finding that triclabendazole induced apoptosis-to-pyroptosis *in vitro*, triclabendazole administration *in vivo* significantly reduced the volume of xenograft tumors by active caspase-3–cleaving protein of GSDME to produce the GSDME-NT fragment. These results revealed that triclabendazole may have therapeutic potential for breast cancer.

Breast cancer is the most common cancer. It is well known that it is heterogeneous with different molecules and prognostic substances, which poses many therapeutic challenges. The current treatment methods include surgery, chemotherapy, radiotherapy, and targeted therapy, but chemotherapy plays a role in anti-cancer activity (Kaufmann & Earnshaw, 2000a). The previous studies revealed that the traditional anti-cancer drugs mainly inhibit the growth and division of tumor cells by interfering with the synthesis of tumor cell DNA and tumor cell microtubule system, leading to tumor cell death (apoptosis and autophagy) and ultimately exerting anti-cancer effects (Kaufmann & Earnshaw, 2000b; Jordan & Wilson, 2004). For example, Taxol induces apoptosis by stabilizing microtubules leading to G2/M cell cycle arrest depending on MAP kinase pathways (ERK and p38) in breast cancer cells (Bacus et al., 2001). Corilagin inhibits breast cancer cell proliferation through inducing apoptosis and autophagy via ROS release (Tong et al., 2018). And unfortunately, approximately only 20% of triple-negative breast cancer responds well to standard chemotherapy (Polyak, 2011). However, recent studies found that inducing secondary necrosis/pyroptosis may be a more effective way to treat cancer (Wang et al., 2017).

Pyroptosis is a new type of programmed cell death, which is mediated by cleaved GSDMD (Shi et al., 2015) and cleaved GSDME (Wang et al., 2017). It has been reported that multiple chemotherapy drugs could promote generation of the active N-terminal fragment of GSDME by active caspase-3 to lead to pyroptosis in cancer cells (Rogers et al., 2017; Wang et al., 2017; Wang et al., 2018; Wu et al., 2019; Yu et al., 2019). Consistent with these studies, we found that triclabendazole treatment induced the GSDME-NT fragment generation and lytic cell death in breast cancer cells which could be partly reversed by caspase-3–specific inhibitor Ac-DEVD-CHO treatment, indicating that the production of GSDME-NT was mediated by active caspase-3. However, besides caspase-3–dependent pyroptosis, some studies indicated that the apoptotic protein caspase-8 induces cleavage of GSDME leading to pyroptosis (Orning et al., 2018; Sarhan et al., 2018). Although we had shown that triclabendazole could induce caspase-8 processing to cleaved caspase-8, our data do not explain whether triclabendazole can directly regulate pyroptosis by activated caspase-8, which indicated further investigation should elucidate the potential pathway underlying the transformation between pyroptosis and apoptosis. In support of *in vitro* results, we also found that administration of triclabendazole significantly induced formation of cleaved caspase-3 and GSDME–N terminal fragment and reduced the volume of the xenograft tumors in the BALB/C nude mice xenograft model. Therefore, these results revealed that triclabendazole induced lytic cell death by caspase-3–mediated GSDME cleavage in breast cancer.

Previous studies have shown that apoptosis can be induced by intrinsic and extrinsic signal pathways, and the active apoptotic caspase will mediate pyroptosis (Zhang et al., 2019). It has also been reported that the intracellular levels of ROS are closely related to apoptosis (Li et al., 2016). When intracellular levels of ROS were overly up-regulated, it could interfere with cellular signaling pathways (Lage et al., 2001). And JNK would play a pivotal role during this process (Lage et al., 2001). Indeed, some studies displayed that phosphorylation of JNK could recruit Bax to mitochondria, leading to the release of cytochrome c followed by caspase-9 cleavage (Lei & Davis, 2003). Concomitantly, the active caspase-9 cleaved caspase-3 to generate the active caspase-3 and induced pyroptosis (Zhou et al., 2018; Yu et al., 2019). Consistent with these studies, in our study, we provided evidence that triclabendazole-induced GSDME-dependent pyroptosis is downstream of the ROS/JNK/Bax-mitochondrial apoptotic pathway. Triclabendazole up-regulated the intracellular levels of ROS and increased the phosphorylation of JNK; it could also up-regulate the expression of Bax and reduce the mitochondrial membrane potential, but these effects of triclabendazole could be counteracted by an ROS scavenger NAC. In support of our findings, another study provided evidence that ROS levels were increased (Zhou et al., 2018), which then facilitates Bax recruitment to mitochondria and gives rise to caspase-3/GSDME-mediated pyroptosis (Wu et al., 2019; Yang et al., 2020). Our results also found that NAC could partly reverse triclabendazole-induced apoptosis-to-pyroptosis. Collectively, these data revealed that triclabendazole induced caspase-3/GSDME-dependent pyroptosis partly at least via the ROS/JNK/Bax signaling pathway. But it is unknown whether there exists other molecular mechanism of caspase-3/GSDME-mediated pyroptosis. Therefore, further investigation is warranted to elucidate the precise mechanism by which triclabendazole induced pyroptosis.

GSDME plays a key role in the pyroptosis process, and the *GSDME* gene is silenced in many human cancer cells including gastric, colorectal, hepatocellular carcinoma, and breast cells (Akino et al., 2007; Wang et al., 2013; Wang et al., 2017). But it is expressed in human breast cancer MCF-7 and MDA-MB-231 cells, indicating they can be targeted to induce secondary necrosis/pyroptosis by drug treatment (Wang et al., 2017). Deficiency of GSDME expression showed resistance to etoposide in melanoma cell lines, but transfecting an expression vector encoding GSDME increased the sensitivity to etoposide (Lage et al., 2001). In lung cancer, genetic deletion of GSDME promoted drug resistance, while GSDME over-expression led to enhanced drug sensitivity *in vivo* and *in vitro* (Lu et al., 2018). Another study reported that knocking out GSDME switches lobaplatin-induced cell death from pyroptosis to apoptosis but does not affect the growth and tumor formation of colon cancer cells treated with lobaplatin (Yu et al., 2019). In our study, triclabendazole-treated breast cells suffered secondary necrosis/pyroptosis with generation of the GSDME-N fragment. Moreover, *in vivo*, we found that triclabendazole administration significantly induced formation of cleaved caspase-3, GSDME-N fragment, and cleaved PARP. At the same time, it also effectively inhibited the growth of the xenograft tumors in the BALB/C nude mice xenograft model. Although we did not explore the effect of triclabendazole on lytic cell death by using deficient GSDME cells, we found that triclabendazole did not induce breast cancer 4T1 cells (deficiency of GSDME expression) to go to secondary necrosis/pyroptosis (without cell swelling and evident large bubbles), which only went to apoptosis (data not shown), thereby suggesting triclabendazole-induced lytic cell death was secondary necrosis/pyroptosis. In addition, in primary gastric cancer and colorectal cancer, GSDME can be suppressed by methylation (Akino et al., 2007; Kim et al., 2008a). We observed that GSDME has low expression in MDA-MB-231 breast cancer cells; at the same time, we also noticed that the ratio of lytic death (annexin V^+^/PI^+^) was lower in MDA-MB-231 cells compared to MCF-7 cells, but we were unclear whether the low expression level of GSDME due to GSDME methylation leads to the lower ratio of lytic death. Some studies also showed that the *GSDME* promoter was also methylated in primary breast cancer cells (Kim et al., 2008b; Croes et al., 2017) and its expression may be up-regulated by treatment of epigenetic drugs, and treatment with DNA methyl-transferase inhibitor decitabine restored GSDME/DFNA5 expression in gastric cancer cell lines (Rogers et al., 2017; Lu et al., 2018), indicating combination with conventional chemotherapy drugs is more effective than each alone, but GSDME expression is a prerequisite. Moreover, GSDME is expressed in normal tissues including the lung, kidney, esophagus, and intestinal epithelium (Wang et al., 2017). And GSDME^−/−^ mice exhibited less adverse effects compared to wild type mice, including tissue damage and weight loss (Rogers et al., 2017). Therefore, in further research, whether the combination therapy can enhance the effect of triclabendazole is worth exploring, and underlying mechanisms of secondary necrosis/pyroptosis require more investigation.

## Conclusion

We found that triclabendazole induced GSDME-dependent pyroptosis by caspase-3 activation at least partly through augmenting the ROS/JNK/Bax-mitochondrial apoptotic pathway. Overall, these findings suggest that triclabendazole promotes pyroptosis in breast cancer cells, representing a promising drug candidate for breast cancer therapy for patients with higher expression of GSDME.

## Data Availability

The original contributions presented in the study are included in the article/Supplementary Material, and further inquiries can be directed to the corresponding authors.
