# Supplementary material for: Triclabendazole Induces Pyroptosis by Activating Caspase-3 to Cleave GSDME in Breast Cancer Cells
Source: Front Pharmacol. 2021 Jul 8;12:670081. doi: 10.3389/fphar.2021.670081 (PMC8297466; doi:10.3389/fphar.2021.670081)
Supplement: Supplementary file 1 [file DataSheet1.docx]

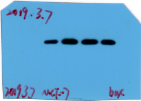

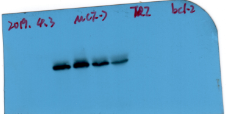

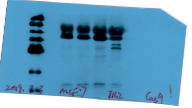


Figure2A Caspase-9 (MCF-7)

Figure2A bcl-2 (MCF-7)

Figure2A bax (MCF-7)


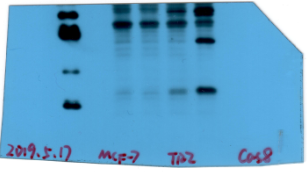

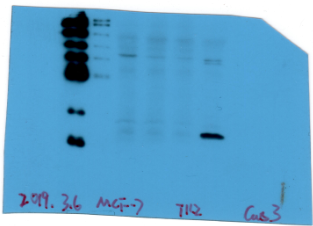

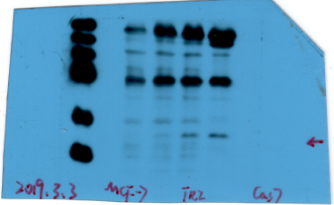


Figure2A Cleaved-cas 3 (MCF-7)

Figure2A Cleaved-cas7(MCF-7)

Figure2A Cleaved-cas8 (MCF-7)


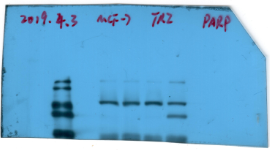

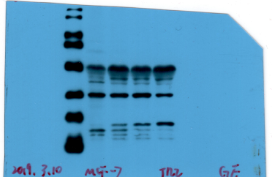

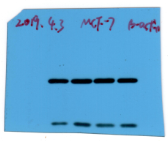


Figure2A β-actin (MCF-7)

Figure2A GSDME (MCF-7)

Figure2A PARP (MCF-7)


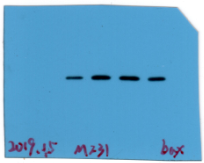

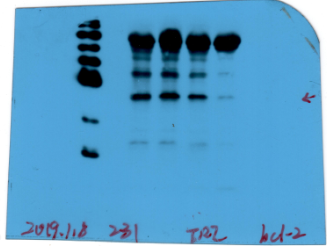

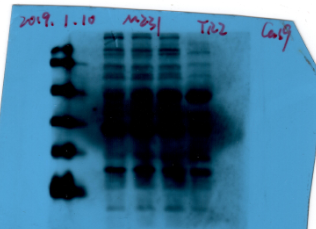


Figure 2B caspase9 (M231)

Figure 2B bcl-2 (M231)

Figure 2B bax (M231)


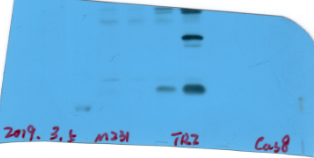

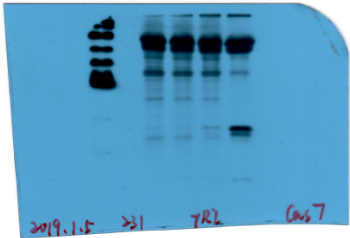

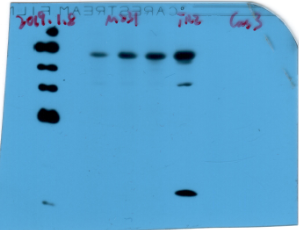


Figure 2B cleaved-cas3 (M231)

Figure 2B cleaved-cas7 (M231)

Figure 2B cleaved-cas8 (M231)


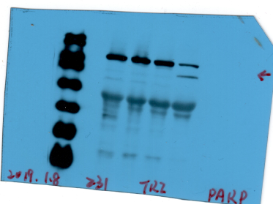

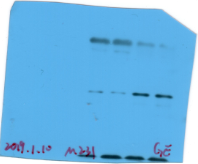

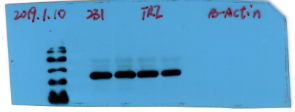


Figure2B β-actin (M231)

Figure 2B GSDME (M231)

Figure 2B PARP (M231)


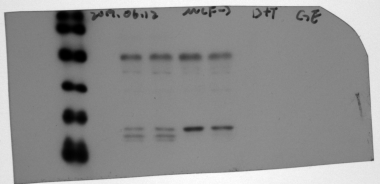

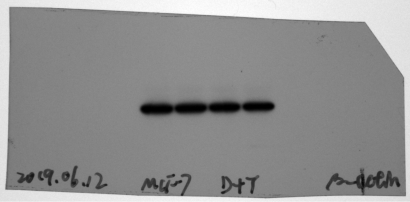

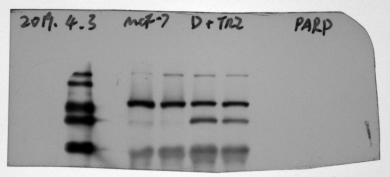


Figure3B GSDME (MCF-7)

Figure3B PARP (MCF-7)

Figure3B β-actin (MCF-7)


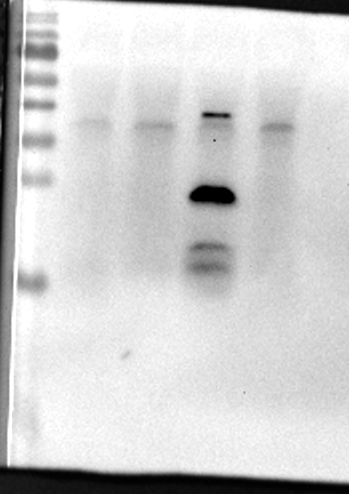

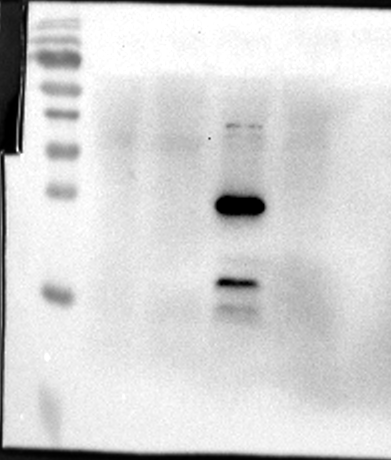

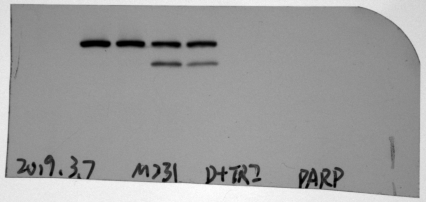


Figure3B PARP (M231)

Figure 3B cleaved-cas3 (MCF-7)

Figure 3B cleaved-cas3 (M231)


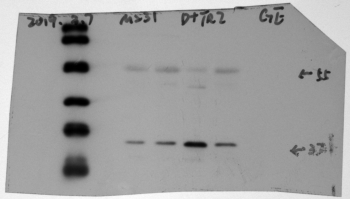

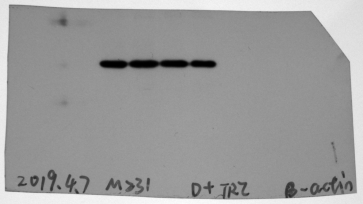

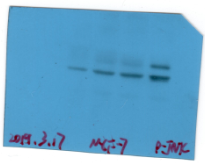


Figure3B β-actin (M231)

Figure3B GSDME (M231)

Figure4C p-JNK(MCF-7)


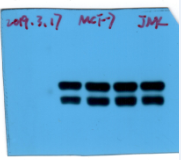

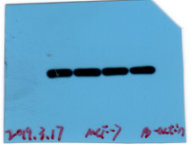

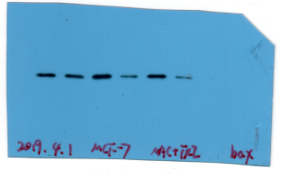


Figure4C bax (MCF-7)

Figure4C β-actin (MCF-7)

Figure4C JNK(MCF-7)


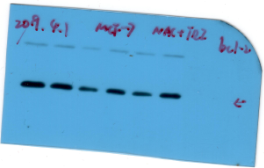

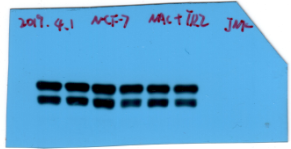

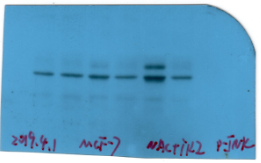


Figure4C JNK (MCF-7)

Figure4C p-JNK (MCF-7)

Figure4C bcl-2 (MCF-7)


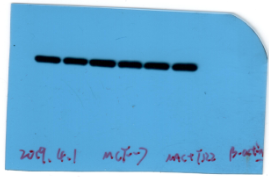

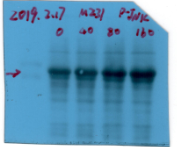

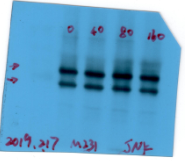


Figure4C JNK (M231)

Figure4C p-JNK (M231)

Figure4C β-actin (MCF-7)


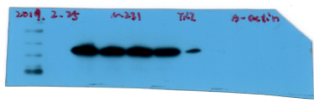

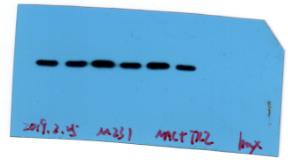

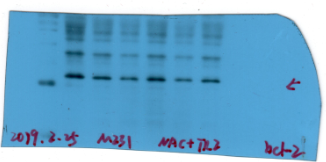


Figure4C bcl-2 (M231)

Figure4C bax (M231)

Figure4C β-actin (M231)


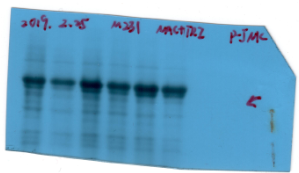

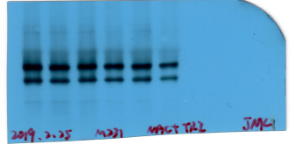

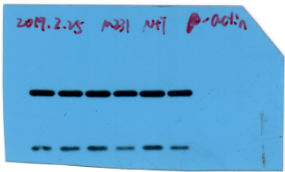


Figure4C β-actin (M231)

Figure4C JNK (M231)

Figure4C p-JNK (M231)


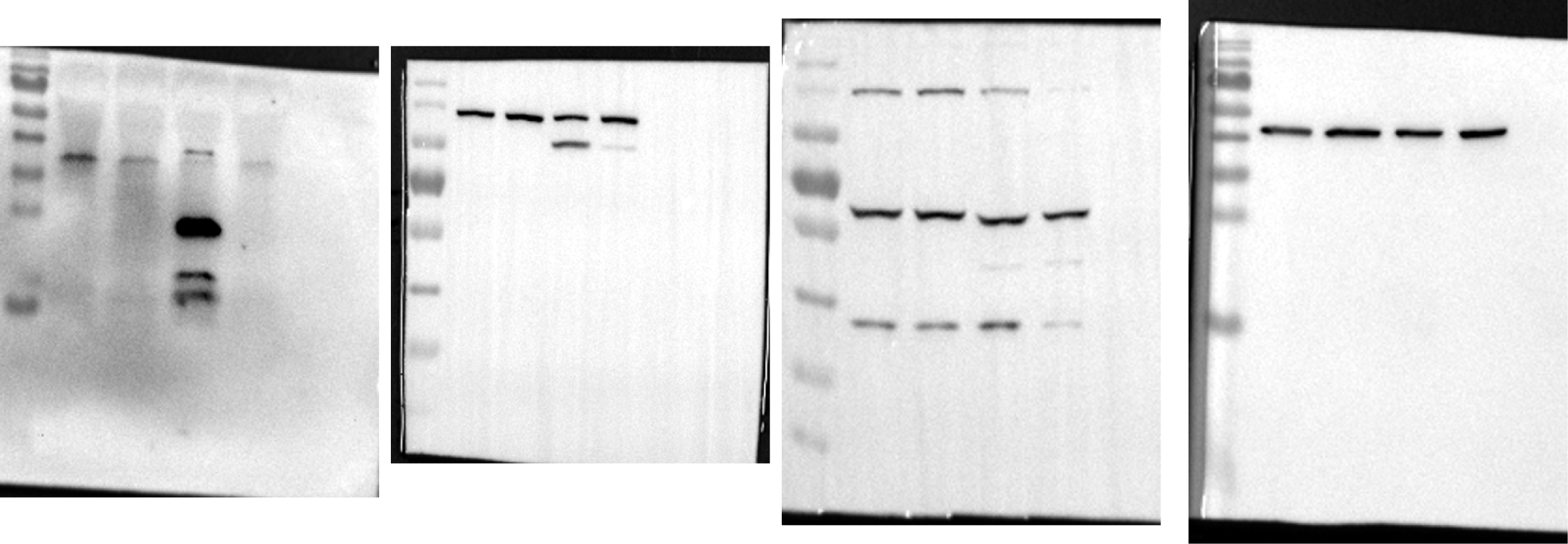


Figure5C β-actin (MCF-7)

Figure 5C GSDME (MCF-7)

Figure 5C PARP (MCF-7)

Figure 5C cleaved-cas3 (MCF-7)


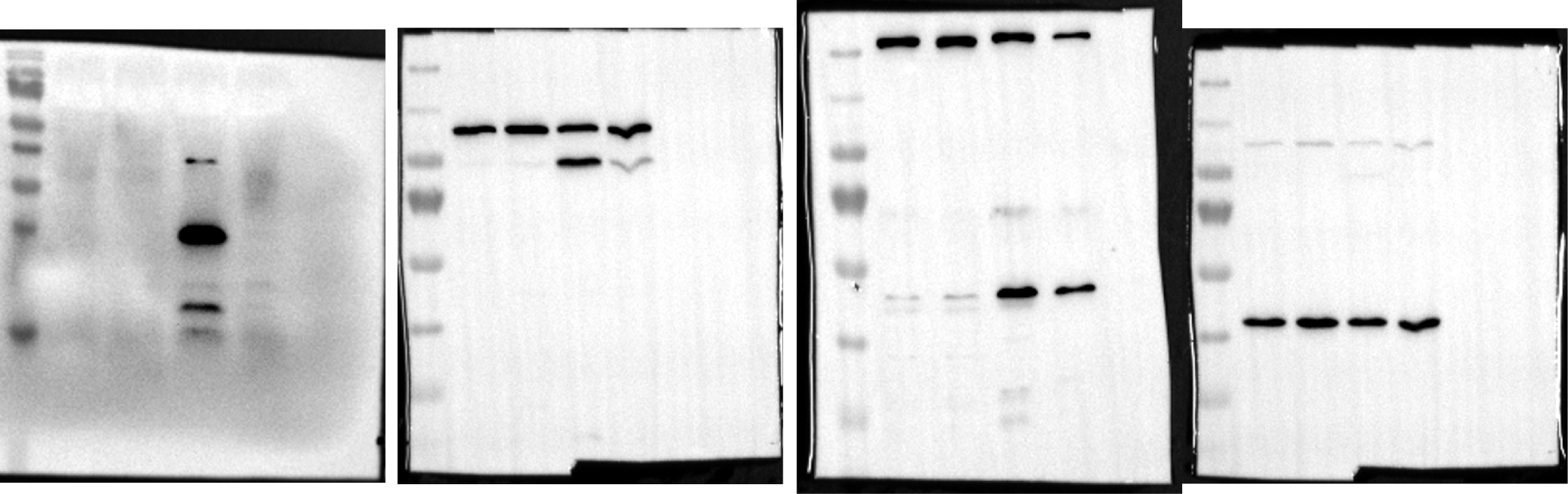


Figure5C β-actin (M231)

Figure 5C GSDME (M231)

Figure 5C PARP (M231)

Figure 5C cleaved-cas3 (M231)


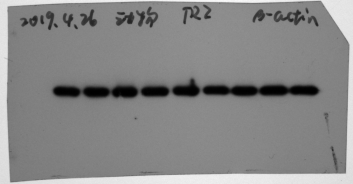

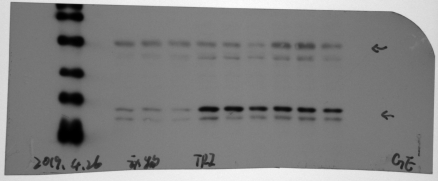

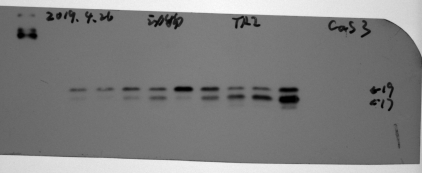


Figure 6D cleaved-cas3

Figure 6D GSDME

Figure6D β-actin


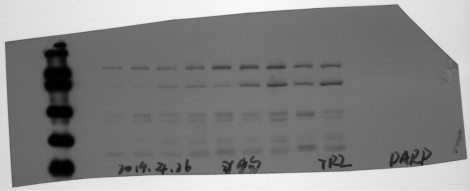


Figure 6D PARP
